# Targeted deprivation of STAT6 sensitizes acute lymphoblastic leukemia cells to cytarabine in vivo and in vitro: clinical implications

**DOI:** 10.1038/s41419-025-07981-7

**Published:** 2025-09-02

**Authors:** Shuzhang Sun, Yixuan Cheng, Xiange Huang, Yinjie Yan, Wanxin Hou, Houshun Fang, Yao Chen, Chunshuang Ma, Yiming Lu, Zhiyi Zhou, Yehuda G. Assaraf, Hui Li, Hegen Li, Ning Xiao

**Affiliations:** 1 Institute of Traditional Chinese Medicine Surgery, Longhua Hospital, Shanghai University of Traditional Chinese Medicine, Shanghai, China; 2 Department of Oncology, Longhua Hospital, Shanghai University of Traditional Chinese Medicine, Shanghai, China; 3 Department of Medical Affairs, Longhua Hospital, Shanghai University of Traditional Chinese Medicine, Shanghai, China; 4 Key Laboratory of Pediatric Hematology and Oncology Ministry of Health, Pediatric Translational Medicine Institute, Shanghai Children’s Medical Center, School of Medicine, Shanghai Jiao Tong University, Shanghai, China; 5 Department of Oncology, Tianshan Hospital of Traditional Chinese Medicine in Changning District, Shanghai, China; 6 The Fred Wyszkowski Cancer Research Laboratory, Faculty of Biology, Technion-Israel Institute of Technology, Haifa, Israel; 7 Fujian Children’s Hospital, Fujian Branch of Shanghai Children’s Medical Center Affiliated to Shanghai Jiao Tong University School of Medicine, Fujian, China

**Keywords:** Translational research, Prognostic markers

## Abstract

Chemotherapy is the leading treatment for acute lymphoblastic leukemia (ALL). However, many ALL patients eventually develop relapses, the treatment of which remains a major challenge due to their chemoresistance phenotype. As a step towards this end, we here uncovered that relapsed ALL specimens exhibit a significantly lower expression of *STAT6* but not of other *STATs*, when compared with their paired diagnosis specimens. Furthermore, STAT6 plays a distinctive role in chemosensitization of ALL cells to cytarabine (Ara-C), and T-box transcription factor 21 *(TBX21)* emerged as a plausible intrinsic biomarker of this Ara-C chemosensitization. We demonstrate that STAT6 undergoes SUMOylation on Lys-307 and sentrin/SUMO-specific protease 3 (SENP3)-mediated deSUMOylation in ALL cells. Most importantly, Ara-C specifically induced SENP3 expression and SENP3 knockdown sensitized ALL cells to Ara-C, with an impact equivalent to STAT6 knockout. These findings support the feedback resistance conferred upon ALL cells by Ara-C-induced SENP3 expression. Our findings uncover a novel role for STAT6 in ALL resistance to Ara-C and suggest that its targeted deprivation or pharmacological inhibition specifically sensitizes ALL cells to Ara-C, offering a plausible modality to surmount Ara-C resistance in future ALL treatment.

## Introduction

Acute lymphoblastic leukemia (ALL) is an aggressive malignancy of lymphoid progenitor cells, and it can invade the bone marrow, blood, and extramedullary sites, with a peak incidence occurring in early childhood and older age [1, 2]. The first-line treatment for ALL typically comprises four phases over 2–3 years, including induction, consolidation, intensification, and long-term maintenance. In this regard, multimodal chemotherapy forms the therapeutic base of ALL therapy [3]. To date, disease-risk stratification and the development of intensified chemotherapy protocols has substantially improved the outcome of ALL patients, particularly children whose 5-year overall survival rate exceeds 90% [4]. However, 15%–20% of ALL patients eventually experience relapses. In addition, the treatment of relapsed ALL remains a true challenge since not only the survival rate lags well behind that observed at initial diagnosis, but the outcome also aggravates at the second or later relapse [5]. Therefore, deciphering the molecular mechanisms underlying chemoresistance and the development of novel modalities to surmount chemoresistance is of paramount importance in the future treatment of ALL.

The protein family of signal transducer and activator of transcription (STAT) comprises seven structurally and functionally related members: STAT1, STAT2, STAT3, STAT4, STAT5a, STAT5b, and STAT6 [6]. Upon stimulation by specific extracellular signals, for instance, cytokines and growth factors, STATs promote the rapid transcription of target genes through a canonical paradigm. Specifically, the latent cytoplasmic STATs become rapidly tyrosine-phosphorylated by Janus kinases after receptor activation, which then facilitates their dimerization and subsequent nuclear translocation to activate gene expression [7]. A growing number of studies have also uncovered noncanonical STAT functions that lie outside this canonical paradigm, such as gene repression, non-nuclear roles, and functions independent of tyrosine phosphorylation [8]. Individual STAT gene ablations corroborated their essential physiological roles, particularly in blood and immune cell, and various functions in mammopoiesis, lactation, postnatal growth, and homeostatic processes [9–11]. In contrast, the potential role of STATs in relapsed ALL remains poorly understood. In the current study, relapsed ALL specimens exhibited a significantly lower expression of *STAT6* but not of other *STATs*, when compared with the paired diagnosis specimens. Moreover, knockout or pharmacological inhibition of STAT6 specifically sensitized ALL cells to the central anti-leukemia drug cytarabine (Ara-C) and increased the incidence of Ara-C-induced apoptosis without altering the normal growth of ALL cells. RNA-Seq results indicate that T-box transcription factor 21 (*TBX21*) can serve as an intrinsic indicator implicated in this Ara-C sensitization of ALL cells. Hence, targeting STAT6 readily sensitizes ALL cells to Ara-C. In this respect, we further found that the anticancer drug monomer Tanshinone Ⅰ (TS1), a natural o-quinone [12], synergized with Ara-C to induce apoptosis in ALL cells by reducing STAT6 levels; this synergistic effect was directly proportional to the immediate status of *TBX21* transcription. Furthermore, we show for the first time that STAT6 underwent SUMOylation on Lys-307 and sentrin/SUMO-specific protease 3 (SENP3)-mediated deSUMOylation in ALL cells. Importantly, Ara-C specifically induced SENP3 expression in ALL cells, and SENP3 knockdown markedly sensitized ALL cells to Ara-C with an impact equivalent to STAT6 targeting. This finding supports the feedback resistance conferred upon ALL cells by Ara-C-induced SENP3. Collectively, our findings reveal a novel role of STAT6 in ALL cell sensitivity to Ara-C and demonstrate the effectiveness of multiple strategies against STAT6, offering a plausible modality to treat ALL via STAT6 targeting.

## Materials and Methods

### Cell culture and transfection

The human ALL cell lines REH, 697, and NALM6 were cultured in RPMI-1640 medium (Gibco, Thermo Fisher Scientific, Waltham, MA, USA). Human embryonic kidney 293T (HEK293T) cells were cultured in DMEM (Gibco). All media were supplemented with 10% fetal bovine serum (Gibco), 100 U/mL penicillin (Thermo Fisher Scientific), and 0.1 mg/mL streptomycin (Thermo Fisher Scientific). Cells were incubated at 37 °C under 5% CO_2_. Cell lines were regularly authenticated and tested for mycoplasma contamination. Transfections were conducted by FuGENE 6 (Promega, Madison, WI, USA) and jetPRIME (Polyplus-transfection, Illkirch, France) according to the manufacturer’s instructions.

### Generation of knockout/knockdown/stable cell lines

Construction of a knockout cell line has been described previously [13]. Briefly, the CRISPR/Cas9 system was used to knockout genes. sgRNA was designed based on the information available at http://crispr.mit.edu and cloned into a vector by following the Zhang laboratory’s protocol. The sgRNA sequences were as follows: STAT6-1#, 5’-CCCTCACCAGGTTCTTGAAC-3’; STAT6-2#, 5’- CATCAACAACACTGTGCCCT-3’; TBX21-1#, 5’-CACCGGTTGTGGCCAAGTTTAATC-3’; TBX21-2#, 5’-CACCGCTACAGGATGTTTGTGGACG-3’. Construction of knockdown or stably-expressed cell lines was described in our previous study [14]. Cells were infected with lentivirus and selected with l μg/mL puromycin (Thermo Fisher Scientific).

### Cell viability assay

The cell viability assay has been described previously [15]. Briefly, cells were seeded in 96-well plates (1.5×10^4^ cells/well) and treated with increasing drug concentrations for 72 h. Cell viability was determined by CellTiter-Glo Luminescent Kit (Promega) according to the manufacturer’s instructions. The half maximal inhibitory concentration (IC_50_) was calculated with GraphPad Prism software. For the growth curve, cells were seeded in 96-well plates (1 × 10^4^ cells/well), and cell viability was detected by CellTiter-Glo Luminescent Kit (Promega) once a day for 5 days. The relative growth rate at different time points was calculated with day 0 as the control.

### Mouse xenograft model

Xenograft models of human ALL were established in B-NDG (NOD-*Prkdc*^*scid*^ *IL2rg*^*tm1*^/Bcgen) mice (Biocytogen, Beijing, China). Parental REH, STAT6 knockout or SENP3 knockout REH cells (1.5 × 10^7^ cells/mouse) were injected into the tail vein of female NDG mice aged 6-8 weeks. After approximately 2 weeks, mice were intraperitoneally treated with Ara-C (500 mg/kg for 2 days), and then mice were sacrificed, bone marrow cells were isolated from the tibia bone, and CD19-positive cells were analyzed by flow cytometry. Animal studies were approved by the Institutional Animal Care and Use Committee of Shanghai Children’s Medical Center.

### Real-time PCR

Total cellular RNA was extracted using Trizol reagent (TIANGEN, Beijing, China). One μg of total RNA was used for reverse transcription into cDNA by the PrimeScriptTM RT reagent kit with cDNA Eraser (Takara, Japan). Q-PCR reactions were performed with SYBR green reagent (Takara, Japan) by a real-time PCR thermocycler (Agilent, USA). Primers used for Q-PCR were as follows: *STAT6*, forward-CCACTTTCAGACAAATACTTCA, reverse-GAGTTCTTCCTGCTTCCA; *TBX21*, forward-TTCCAACACGCATATCTT, reverse-AGTAATCTCGGCATTCTG; *FRK*, forward-AATGCCTTACAGTGGTATGA, reverse-GATGGTTGCGGAAGTCTA; *GPRIN3*, forward-CCTACTGCTCAATCCTAA, reverse-CTCCTGGTTCTTCCTAAT; *GAPDH*, forward-GAGCTGAACGGGAAGCTCACTG, reverse-TGGTGCTCAGTGTAGCCCAGGA.

### Cell apoptosis assay

Cells were seeded in 12-well plates (1 × 10^5^ cells/well) and incubated with the indicated drugs for 72 h. Cells were then harvested for staining by Annexin V apoptosis detection kit (Elabscience, China), and the percentage of Annexin V-positive cells was determined using a FACSCalibur flow cytometer (BD Biosciences, USA).

### Immunoprecipitation and Western blot analysis

The immunoprecipitation and Western blotting protocols have been described previously [16]. Immunoprecipitates and cell lysates were analyzed by SDS-PAGE and proteins of interest were detected using the following antibodies: STAT6 (ab32520, Abcam, Cambridge, United Kingdom), p-Y641-STAT6 (ab263947), γH2AX (ab81299); H2AX (#7631S, Cell Signaling Technology, Dallas, TX, USA), Cleaved PARP (#5625S), PARP (#9542S), SUMO1 (#4930), SUMO2/3 (#4971), GST-Tag (#2624); Actin (R1207-1, HUABIO) and GAPDH (R1207-1); SENP2 (GTX110504, GeneTex); SENP3 (A303-139A, Bethyl Laboratories, Montgomery, TX, USA); FLAG M2 Affinity Gel (A2220, MilliporeSigma, Burlington, MA, USA) and anti-FLAG M2 (F3165); HA-Tag (66006-1-Ig, Proteintech) and Myc-Tag (60003-2-Ig). Immunoblots were imaged and analyzed using the Odyssey system (LI-COR Biosciences, USA), ImageQuant LAS4000 (GE Healthcare, USA), or ChemiDoc MP (BioRad, USA).

### Prokaryotic SUMO-conjugation system

The prokaryotic SUMOylation system was previously described [15, 17]. Briefly, GST-tagged recombinant plasmids pGEX-STAT6-WT/K307R/K621R were separately co-transfected with pE1E2S1 (or pE1E2S2) into *Escherichia coli* BL21 (DE3) competent cells and then grown on the plate coated with chloramphenicol and ampicillin LB. The colonies were transferred to liquid LB medium, and 0.2 mM isopropyl-β-D-thiogalactopyranoside was added when OD_600_ reached 0.6. Next, the bacterial suspension was incubated for 12 h at 25 °C and then harvested for the purification of SUMOylated proteins by GST-pull down assay.

### RNA sequencing

Cells were seeded in 12-well plates (1 × 10^5^ cells/well), and lysed with Trizol reagent. Establishment of cDNA library, RNA sequence analysis, quality control,l and transcriptome profiling were performed by TIANGEN Biotech Co., Ltd (Beijing, China). Details can be found on the official website: https://www.tiangen.com.

### Quantification and statistical analysis

Sample sizes and reproducibility for each figure are denoted in the figure legends. Statistical analysis and associated statistical graphics were established by GraphPad Prism software. Statistical significance between conditions was calculated using two-tailed Student’s t-tests, NS: *p* > 0.05; *p < 0.05; **p < 0.01; ***p < 0.001. Error bars denote the S.D. All statistical details can be found in the methods and/or figure legends.

## Results

### STAT6 deprivation sensitizes ALL cells to Ara-C

**Fig. 1:**
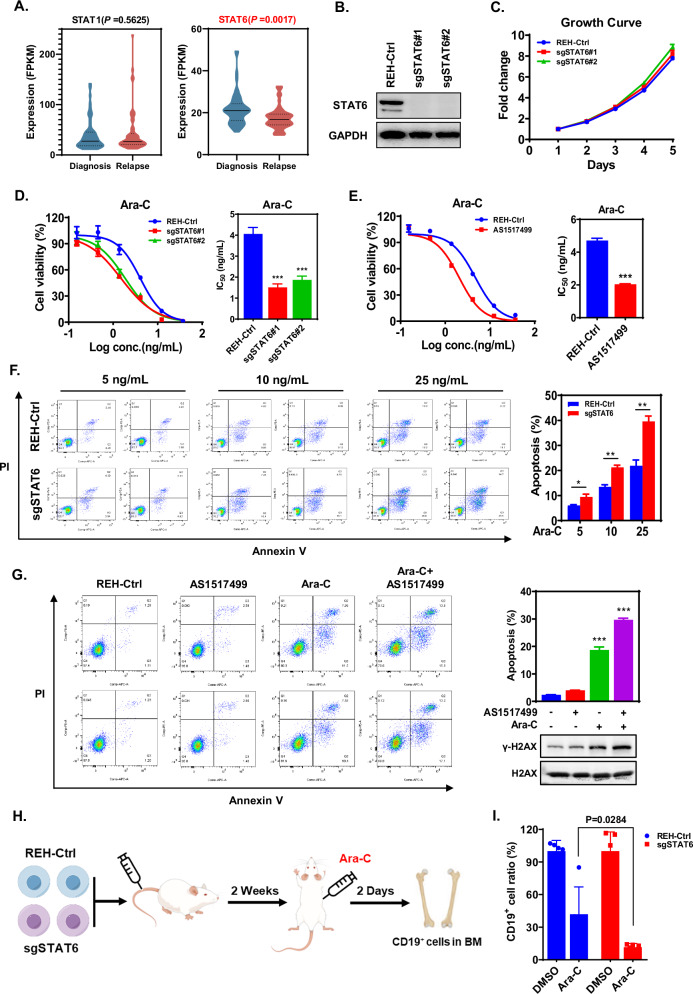
STAT6 deprivation sensitizes ALL cells to Ara-C. **A** Gene expression of STAT1 and STAT6 using RNA-Seq analysis of 53 paired diagnosis-relapse ALL specimens. Two sets of data are compared using two-tailed Student’s *t*-test, with *p* < 0.05 as a statistically significant difference. **B** Endogenous STAT6 in REH cells was knocked out using CRISPR-Cas9 technology. **C** Growth curve of parental REH and STAT6-knockout REH cells. Data are presented as mean ± SD. **D** Ara-C chemosensitivity of parental REH and STAT6-knockout REH cells as determined by the cell viability assay. Data are presented as a dose-response curve (left panel) and bar graph of IC_50_ values (right panel). Each group is compared with the control using a two-tailed Student’s *t*-test, ***p < 0.001. **E** Ara-C chemosensitivity of REH cells (or REH cells pretreated with 10 nM AS1517499) to Ara-C as determined by the cell viability assay. Data are presented as a dose-response curve (left panel) and bar graph of IC_50_ values (right panel), two-tailed Student’s *t*-test, ***p < 0.001. **F** The rate of apoptosis in parental REH or STAT6-knockout REH cells treated with increasing concentrations of Ara-C and analyzed by flow cytometry. The quantitative bar graph is shown on the right and data are depicted as mean ± SD, *p < 0.05; **p < 0.01. **G** Ara-C (20 ng/mL) induces apoptosis in REH cells or REH cells pretreated with 10 nM AS1517499. The quantitative bar graph and detection of apoptotic biomarker are shown on the right. Data are presented as mean ± SD, each group is compared with the control using two-tailed Student’s *t*-test, ***p < 0.001. Schematic diagram showing the construction and drug treatment strategy of mouse xenograft model (**H**) and the number of CD19^+^ REH cells isolated from the bone marrow (BM) of mice as analyzed by flow cytometry (**I**). Data are presented as mean ± SD.

To assess the potential roles of STATs in relapsed ALL, we re-analyzed RNA-Seq data from 53 matched pairs of diagnosis-relapse ALL specimens [18]. The results reveal a significantly lower expression of *STAT6* but not of other *STATs* in relapsed specimens than in diagnosis specimens (Fig. 1A and Supplementary Fig. 1A). This finding raised the question of whether STAT6 plays specific roles in ALL relapse. Towards this end, we firstly deprived the human ALL cell line REH of its endogenous STAT6 using CRISPR-Cas9 knockout to assess the possible impact of STAT6 on the chemosensitivity of ALL cells (Fig. 1B). Notably, upon treatment with Ara-C, the viability of STAT6-knockout REH cells markedly decreased when compared with the control cells (Fig. 1D). Remarkably, these cells presented minimal differences from the control cells after treatment with other common chemotherapeutic drugs used for ALL therapy, including methotrexate (MTX), 6-mercaptopurine (6-MP), vincristine (VCR), L-asparaginase (L-Asp), and daunorubicin (DNR) (Supplementary Fig. 1B–F). Consistently, similar results were obtained with the other two ALL cell lines, NALM6 and 697, hence corroborating the enhanced chemosensitivity of ALL cells to Ara-C via STAT6 knockout (Supplementary Fig. 1G–I). Furthermore, pharmacological inhibition of STAT6 by AS1517499, a specific inhibitor that abolishes the tyrosine phosphorylation and nuclear translocation of STAT6 [19], also sensitized ALL cells to Ara-C (Fig. 1E). The cytotoxic activity of various chemotherapeutic drugs were previously ascribed to their capability to induce genotoxicity and apoptosis [20]. Indeed, STAT6 knockout or pharmacological inhibition substantially promoted Ara-C-induced apoptosis of ALL cells (Fig. 1F and G and Supplementary Fig. 1J–L), without altering their normal growth (Fig. 1C and Supplementary Fig. 1M). To validate the implications of STAT6 knockout in vivo, we used a mouse leukemia xenograft model by injecting parental REH or STAT6-knockout REH cells into NDG mice (Fig. 1H) and observed no significant impact of STAT6 knockout on leukemogenesis in vivo (Fig. 1I). In contrast, Ara-C treatment was significantly superior in eradicating STAT6-knockout REH cells rather than their parental REH cells (Fig. 1I), indicating that STAT6 knockout markedly enhances the sensitivity of ALL cells to Ara-C in vivo. Altogether, these findings reveal that STAT6 deprivation markedly sensitizes ALL cells to Ara-C in vitro and in vivo.

### *TBX21* serves as an indicator of tg-STAT6-enhanced sensitivity of ALL cells to Ara-C

**Fig. 2:**
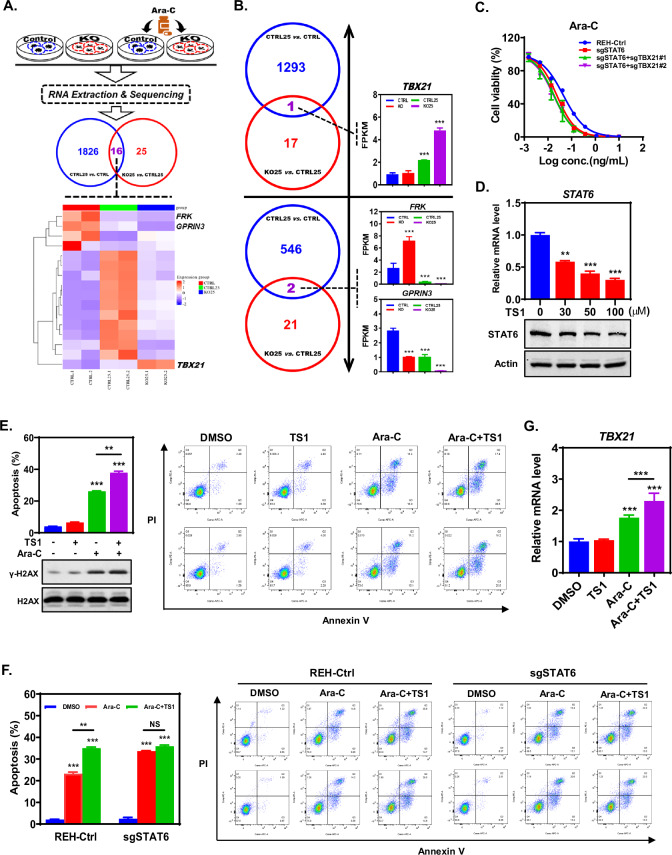
*TBX21* serves as an indicator of tg-STAT6-enhanced sensitivity of ALL cells to Ara-C. **A** Schematic depiction of the RNA sequencing strategy of the indicated REH cells and the intersection of differentially expressed genes (DEGs) between ‘Cluster Ⅰ’ and ‘Cluster Ⅱ’ was displayed via a graphical heatmap. Parental REH or STAT6-knockout REH cells were treated with or without 25 ng/mL Ara-C. **B** The intersection of upregulated DEG (*TBX21*) or downregulated DEGs (*FRK* and *GPRIN3*) between ‘Cluster Ⅰ’ and ‘Cluster Ⅱ’, the histograms of their relative expression are shown on the right. Data are presented as mean ± SD, each group is compared with the control using a two-tailed Student’s *t*-test, ***p < 0.001. **C** Knockout of *TBX21* does not counteract the enhanced chemosensitivity of ALL cells to Ara-C caused by STAT6 knockout. The chemosensitivity of the indicated REH cells to Ara-C is determined by the cell viability assay. **D** Effect of increasing concentrations of TS1 on the mRNA (12 h) and protein levels (24 h) of STAT6 in REH cells. Each group is compared with the control using a two-tailed Student’s *t*-test, **p < 0.01; ***p < 0.001. **E** TS1 (10 mM, 48 h) synergizes with Ara-C (20 ng/mL) to induce apoptosis in REH cells. The quantitative bar graph and the detection of apoptotic biomarker are shown on the left. Data are presented as mean ± SD, each group is compared with the control using two-tailed Student’s *t*-test, **p < 0.01; ***p < 0.001. **F** The synergistic effect of TS1 on Ara-C-induced apoptosis in REH cells is dependent on STAT6. The quantitative bar graph is shown on the left. Data are presented as mean ± SD, two-tailed Student’s *t*-test, **p < 0.01; ***p < 0.001. **G** Relative mRNA levels of *TBX21* in REH cells and REH cells pretreated with TS1 (10 mM, 48 h) under the treatment of Ara-C (20 ng/mL) as determined by real-time quantitative PCR (RT-qPCR). Data are presented as mean ± SD, each group is compared with the control using two-tailed Student’s *t*-test, ***p < 0.001.

STAT6 belongs to a family of transcription factors that transmits signals from a receptor complex to the nucleus, triggering transcriptional regulation after recruitment of multifarious cofactors to the transcriptosome [6]. We therefore performed RNA sequencing of REH cells to explore the mechanism underlying STAT6 knockout in the chemosensitization of ALL cells to Ara-C at the transcriptional level (Fig. 2A). Given that STAT6 knockout can markedly enhance the chemosensitivity of REH cells to Ara-C and promote Ara-C-induced apoptosis, we first screened out the differentially expressed genes (DEGs) of ‘KO25 *vs* CTRL25’, referred to as ‘Cluster Ⅰ’, for STAT6-knockout-mediated enhancement of the proapoptotic effect of Ara-C. We next gathered the DEGs of ‘CTRL25 *vs* CTRL’, referred to as ‘Cluster Ⅱ’, for Ara-C acting on REH cells. Theoretically, the intersection between ‘Cluster Ⅰ’ and ‘Cluster Ⅱ’ probably masked our target. Based on this rationale, we identified 16 candidate target genes and displayed their relative expression levels via a graphical heatmap (Fig. 2A). Of these genes, *TBX21* displayed a consistent up-regulation, whereas *FRK* and *GPRIN3* were down-regulated during the comparison of ‘Cluster Ⅰ’ with ‘Cluster Ⅱ’ (Fig. 2B). Moreover, complete analysis of the relative expression of these three genes suggested a possible role for *TBX21* in the sensitization of ALL cells to Ara-C via STAT6 knockout (Fig. 2B). However, *TBX21* knockout did not rescue the enhanced sensitization of ALL cells to Ara-C caused by STAT6 knockout (Fig. 2C). This suggests that *TBX21* was likely an ‘indicator’ rather than a ‘driver’ throughout the targeting of STAT6 (tg-STAT6) that sensitized ALL cells to Ara-C. We therefore screened a small compound library composed of anticancer monomers of Traditional Chinese Medicine (TCM) and identified a drug monomer called TS1, which downregulated STAT6 expression (Supplementary Fig. 2A). TS1 specifically reduced *STAT6* gene expression in ALL cells in a time- and dose-dependent manner (Fig. 2D and Supplementary Fig. 2A–D). Given the capacity of TS1 to significantly reduce STAT6 gene expression in ALL cells, TS1 may affect ALL cells through its interplay with Ara-C. To test this hypothesis, we pretreated REH (or 697) cells with TS1 to assess Ara-C-induced apoptosis. The results demonstrated that TS1 synergized with Ara-C to induce ALL cell apoptosis and this synergism was tightly dependent on STAT6 (Fig. 2E and F and Supplementary Fig. 2E). Moreover, *TBX21* mRNA levels were directly proportional to the synergistic effect of TS1 with Ara-C (Fig. 2G and Supplementary Fig. 2F), which not only echoes the above RNA-Seq data but also establishes the specific role of *TBX21* in tg-STAT6-enhanced chemosensitization of ALL cells to Ara-C.

### STAT6 is SUMOylated on Lys-307

**Fig. 3:**
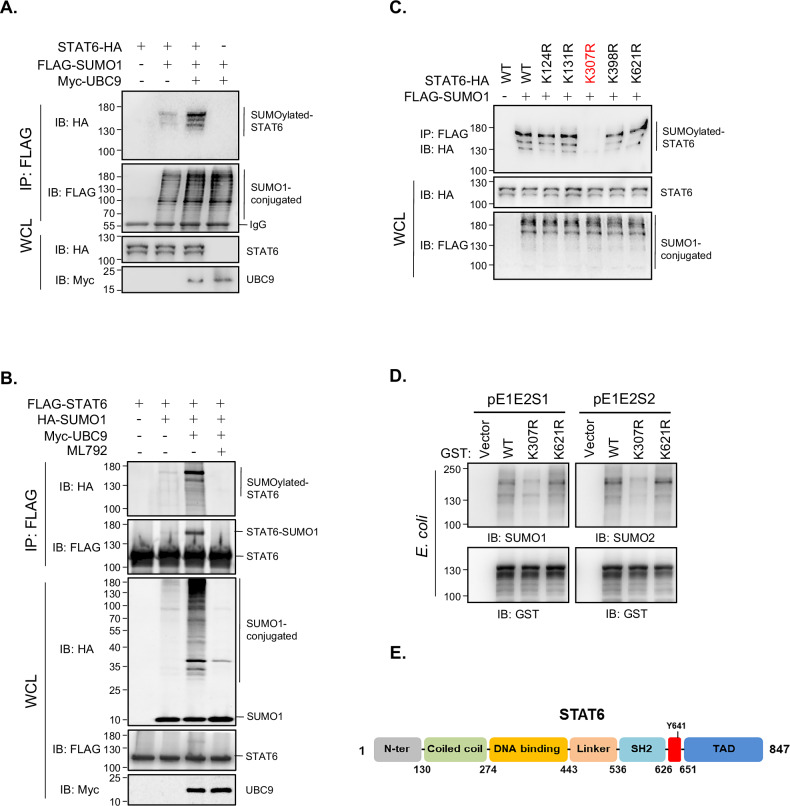
STAT6 is SUMOylated on Lys-307. **A**, **B** STAT6 is SUMOylated in cultured human cells. HEK293 cells were transfected with the indicated plasmids and harvested at 48 h after transfection, followed by immunoprecipitation (IP) and Western blot (WB) analysis. WCL: whole cell lysate. **C** Lys-307 emerges as a critical SUMO-acceptor site of STAT6. HEK293 cells were transfected with the indicated plasmids and harvested at 48 h after transfection, followed by IP and WB analysis. **D** STAT6 undergoes SUMOylation on Lys-307 as further confirmed by the prokaryotic SUMO-conjugation system. **E** Schematic diagram of the structural domains of the STAT6 protein.

Given the extensive regulation of the STAT family of transcription factors via SUMOylation [21–24], we investigated whether STAT6 can also be modified by SUMOylation. We performed a in vivo SUMOylation assay in HEK293 cells with the cotransfection of exogenously-tagged STAT6 and/or SUMO1 with (or without) Myc-UBC9. We observed that UBC9, which is the sole SUMO-specific E2-conjugating enzyme, markedly enhanced STAT6 SUMOylation (Fig. 3A, B). In contrast, ML792, a SUMO-activating enzyme inhibitor, abolished STAT6 SUMOylation (Fig. 3B, lane 4 *vs*. lane 2/3). Based on the *SUMOplot*^*TM*^ *Analysis Program*’s prediction of candidate motifs with high probabilities of SUMO conjugation, we individually mutated the putative SUMO-acceptor Lys to Arg on STAT6 and observed that Lys-307 to Arg-307 (K307R) substitution markedly reduced STAT6 SUMOylation, whereas other mutations failed to do so (Fig. 3C). Furthermore, the results from a prokaryotic SUMO-conjugation system confirmed STAT6 as a novel SUMOylation target and Lys-307 as a critical SUMO-acceptor site on STAT6 (Fig. 3D). We also noted that STAT6 can undergo SUMO2-conjugation on Lys-307 (Fig. 3D), a lysine residue located in the DNA-binding domain (Fig. 3E), which suggests the potential importance of regulating SUMOylation in STAT6 function.

### SENP3 targets STAT6 for deSUMOylation in ALL cells

**Fig. 4:**
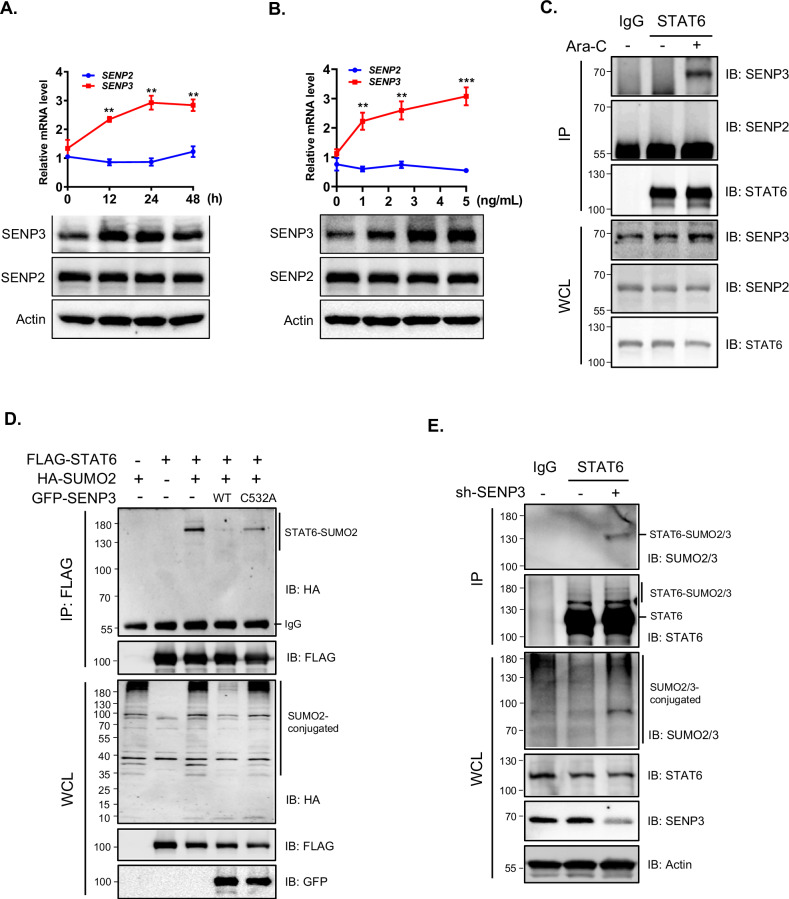
SENP3 targets STAT6 for deSUMOylation in ALL cells. **A**, **B** Specific induction of SENP3 in REH cells by Ara-C. REH cells were treated with Ara-C for the indicated time and dosage, and then harvested for RT-qPCR or WB analysis. Data are presented as mean ± SD, two-tailed Student’s *t*-test, **p < 0.01; ***p < 0.001. **C** Ara-C induces specific association of STAT6 with SENP3 in REH cells. Whole cell lysates were immunoprecipitated using an anti-STAT6 antibody and then analyzed by WB analysis. **D** SENP3 deSUMOylates SUMO2-conjugated STAT6 in cultured human cells. HEK293 cells were transfected with the indicated plasmids and harvested at 48 h after transfection, followed by IP and WB analysis. **E** SUMO2/3-conjugated STAT6 accumulates in REH cells upon SENP3 knockdown. Endogenous SENP3 was stably knocked down in REH cells and then analyzed by IP and WB analysis.

Interestingly, Ara-C specifically induced the expression of the SUMO-specific protease *SENP3* (but not *SENP2*) in REH cells, and this induction of SENP3 was time- and dose-dependent upon Ara-C treatment (Fig. 4A, B). In addition, Ara-C promoted the association of STAT6 with SENP3 in REH cells (Fig. 4C and Supplementary Fig. 3A). We further demonstrated the SUMO2-conjugated SUMOylation of STAT6, and that wild type SENP3 (SENP3^-WT^), but not the catalytic dead SENP3 mutant (SENP3^-C532A^), readily mediated the deSUMOylation of STAT6 (Fig. 4D). Moreover, SUMO2/3-conjugated SUMOylation of STAT6 significantly accumulated in SENP3 knockdown REH cells (Fig. 4E). These results indicate that that SENP3 can target STAT6 for deSUMOylation in ALL cells.

### Ara-C-induced SENP3 confers a feedback resistance upon ALL cells

**Fig. 5:**
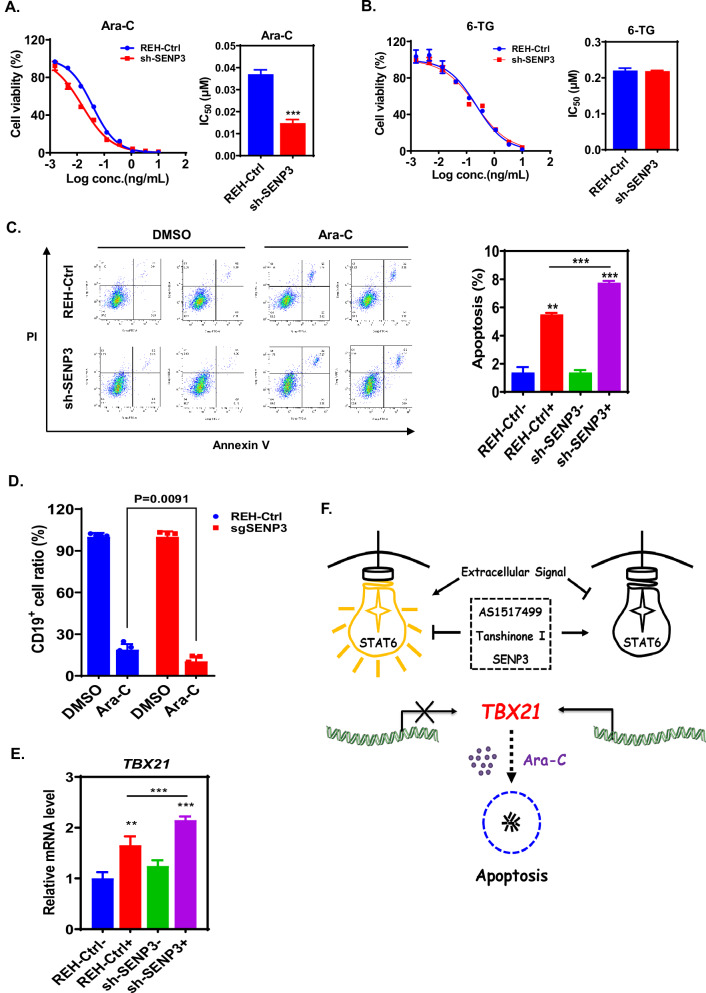
Ara-C-induced SENP3 confers a feedback resistance upon ALL cells. **A** SENP3 knockdown sensitizes REH cells to Ara-C as determined by the cell viability assay. Data are presented as dose-response curve (left panel) and bar graph of IC_50_ values (right panel), two-tailed Student’s *t*-test, ***p < 0.001. **B** SENP3 knockdown does not affect the chemosensitivity of cells to 6-TG as determined by the cell viability assay. Data are presented as mean ± SD and the quantitative bar graph is shown at the lower panel. **C** SENP3 knockdown promotes Ara-C-induced apoptosis in REH cells as determined by flow cytometry. Data are presented as mean ± SD and the quantitative bar graph is shown at left panel, two-tailed Student’s *t*-test, **p < 0.01; ***p < 0.001. **D** SENP3 knockdown augments the chemosensitivity of ALL cells to Ara-C in vivo. The number of CD19^+^ REH cells from the BM of mice bearing ALL xenografts was determined by flow cytometry. Data are presented as mean ± SD. **E** Relative mRNA levels of *TBX21* in parental REH and SENP3-knockdown REH cells under the treatment of Ara-C (20 ng/mL) as determined by RT-qPCR. Data are presented as mean ± SD, two-tailed Student’s *t*-test, **p < 0.01; ***p < 0.001. **F** A schematic model for the chemosensitization of ALL cell to Ara-C via STAT6 targeting.

Regarding the chemosensitization of ALL cells to Ara-C via STAT6 targeting, we observed that cells with SENP3 knockdown displayed an enhanced sensitivity to Ara-C but not to 6-TG (Fig. 5A, B and Supplementary Fig. 3B, C). Moreover, SENP3 knockdown increased the susceptibility of ALL cells to Ara-C-induced apoptosis and enhanced the sensitivity of ALL cells to Ara-C in vivo (Fig. 5C, D and Supplementary Fig. 3D). In particular, the transcriptional status of *TBX21* further revealed the specific role of SENP3 in the chemosensitization of ALL cells to Ara-C via STAT6 targeting (Fig. 5E and Supplementary Fig. 3E), hence supporting the notion that Ara-C-induced SENP3 confers a feedback resistance upon ALL cells at least to some extent.

## Discussion

All STAT proteins contain six common conserved domains that mediate various aspects of STAT function: N-terminal domain, coiled-coil domain, DNA-binding domain, linker domain, Src homology-2 domain, and transactivation domain (TAD) [6]. The specificities of STATs are influenced by divergent TAD, tissue-specific expression and multiple receptor-activated signaling; in addition, dysregulation of STATs often leads to immune system disorders and the development of various malignancies [6, 25]. STAT6, a key member of the STAT family, can be stimulated primarily by the cytokines IL-4 or IL-13; it acts as a T helper type 2 (Th2)-inducing transcriptional activator in tuning epigenetic modifications and orchestrating the maturation of peripheral Th2 cells [25]. In addition, other cytokines, such as IL-3/15, interferon-α, and platelet-derived growth factor (PDGF), activate STAT6 in diverse cell lines and function in nonimmune systems and the pathogenesis of several diseases, including allergic responses, lymphomas, and leukemias [7, 26]. Based on these findings, targeting STAT6 may be an attractive modality for the treatment of these malignant and non-malignant disorders. In the current study, we unveiled a distinctive role of STAT6 in chemosensitization of ALL cells to Ara-C via various strategies, including gene knockout, pharmacological inhibition, SUMOylation and downregulation of expression. Our findings reveal that STAT6 targeting specifically sensitizes ALL cells to Ara-C. We also explored the molecular mechanism underlying this Ara-C potentiation: tg-STAT6-enhanced chemosensitization of ALL cells to Ara-C was positively correlated with the transcription of *TBX21*, whose coded protein T-bet serves as a bridge between innate and adaptive immunities by regulating a transcriptional network of genetic programs [27]. In contrast, deoxycytidine kinase (*dCK*), which catalyzes the intracellular activation of Ara-C [28], was not included in these 16 candidate target genes involved in this enhanced Ara-C sensitization. However, our present findings also uncovered that *TBX21* is not the “driver” but rather a marker of tg-STAT6-enhanced chemosensitization of ALL cells to Ara-C, although the knockout or downregulation of STAT6 can considerably lead to an increase of Ara-C-induced transcription of *TBX21*. Regarding the relevance of the possible linkage of *TBX21* (or T-bet) to Ara-C chemosensitization, whether STAT6 can exert noncanonical transcriptional inhibition on *TBX21*, warrants further studies.

ALL is the most common childhood cancer, accounting for over 25% of all types of childhood malignancies [29]. Chemotherapy remains the most effective approach against ALL, albeit several newly targeted therapies and molecularly targeted agents have been developed for ALL treatment [3, 4]. However, relapse remains a leading cause of mortality, and drug resistance is a major impediment in ALL treatment. Therefore, deciphering the molecular mechanisms underlying resistance to various chemotherapeutic agents as well as designing novel strategies to surmount chemoresistance are of paramount importance for curative treatment of ALL. As a step towards this end, we herein first found that relapsed ALL specimens exhibit a substantially lower STAT6 expression compared with their paired diagnosis specimens; this differs from previous studies, which showed a high expression of STAT6 in various tumors and its association with tumorigenesis and poor prognosis in human cancers [26, 30–32]. Thus, notably, targeting STAT6 can specifically enhance the chemosensitization of ALL cells to Ara-C by boosting Ara-C-induced apoptosis; this implies that STAT6 targeting may prove as a novel modality to surmount Ara-C resistance in ALL. In this regard, we verified that TS1, a lipid-soluble phenanthraquinone extracted from the root of TCM *Salvia miltiorrhiza* and displaying favorable activities in antioxidant, anti-inflammatory, anti-cancer as well as plays a role in the regulation of cell autophagy or apoptosis [33], can synergize with Ara-C to induce apoptosis in ALL cells by reducing STAT6 expression. This phenomenon was in direct correlation to the transcription of *TBX21*. In summary, these findings provide not only a potential therapeutic target for overcoming Ara-C resistance but also a novel strategy for the combined application of TCM and Western medicine in the treatment of ALL.

We have also discovered that STAT6 undergoes SUMOylation, which is an essential posttranslational modification considered a similar enzymatic machinery to ubiquitination [34]. In particular, switching on or off SUMOylation involves a dimeric E1 (SAE1/UBA2), a single E2 (UBC9), a limited quantity of E3, and mainly, a deSUMOylase family [35]. The latter comprises six SENPs (SENP1–3 and 5–7), which play decisive roles in SUMO maturation/cycling and protein SUMOylation [36]. However, studies rarely depicted the association between SENPs and ALL. We previously reported that SENP1, rather than SENP2, can specifically mediate the antileukemic effects of topoisomerase Ⅰ inhibitors like camptothecin derivatives and topotecan on ALL. In contrast, neither SENP1 nor SENP2 altered the chemosensitivity of ALL cells to diverse chemotherapeutic drugs [37]. Here, we found that SENP3, which shows a preference for SUMO2/3-conjugated deSUMOylation unlike SENP1/2 [36], targets STAT6 for deSUMOylation in ALL cells. To the best of our knowledge, no previous study reported the relevance of SENP3 in ALL, although SENP3 plays important roles in acute myeloblastic leukemia (AML), promyelocytic leukemia (PML) and osteogenic differentiation of stem cells [38–40]. Most importantly, Ara-C specifically induces SENP3 expression in ALL cells, and SENP3 knockdown considerably sensitizes ALL cells to Ara-C, with a similar impact of targeting STAT6. This suggests that Ara-C-induced SENP3 expression confers upon ALL cells a feedback resistance. Overall, our findings reveal a novel role of STAT6 in ALL cells and validate multiple effective strategies for targeting STAT6 (Fig. 5F) and thus provide potential new avenues for overcoming of Ara-C resistance in future ALL treatment.

## Supplementary information

Supplementary Figure 1-3

Supplementary Figure Legends

Original Data

## Data Availability

The data and materials will be made available on request.
