# Supplementary material for: Targeted deprivation of STAT6 sensitizes acute lymphoblastic leukemia cells to cytarabine in vivo and in vitro: clinical implications
Source: Cell Death Dis. 2025 Sep 2;16(1):669. doi: 10.1038/s41419-025-07981-7 (PMC12405434; doi:10.1038/s41419-025-07981-7)
Supplement: Supplementary file 1 — Supplementary Figure 1-3 [file 41419_2025_7981_MOESM1_ESM.pptx]

## Slide 1
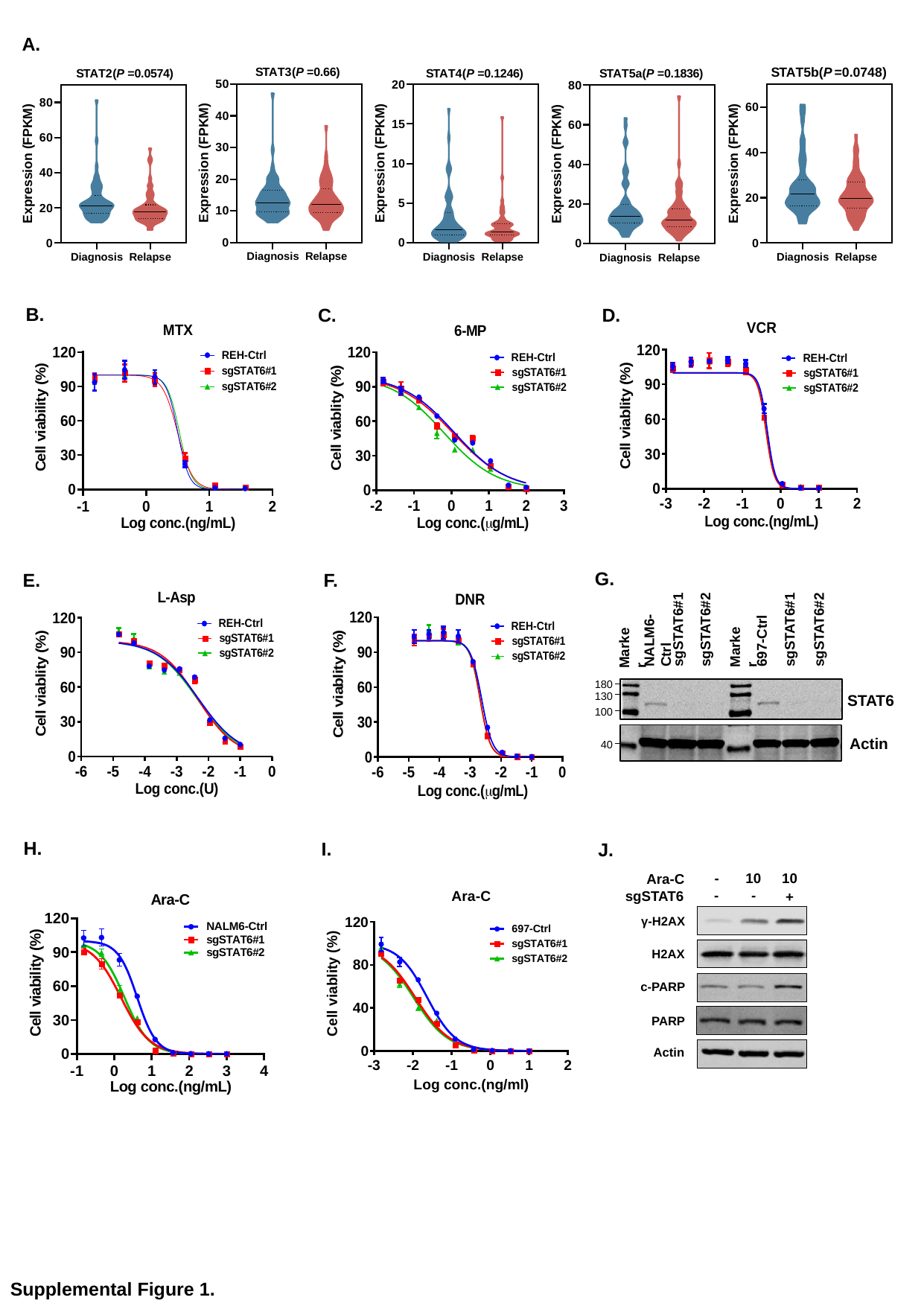

A.
B.
C.
D.
G.
E.
F.
sgSTAT6#2
sgSTAT6#2
NALM6-Ctrl
sgSTAT6#1
sgSTAT6#1
697-Ctrl
Marker
Marker
180
130
STAT6
100
Actin
40
H.
I.
J.
10
10
-
Ara-C
-
-
sgSTAT6
+
 γ-H2AX
H2AX
c-PARP
PARP
Actin
Supplemental Figure 1.

## Slide 2
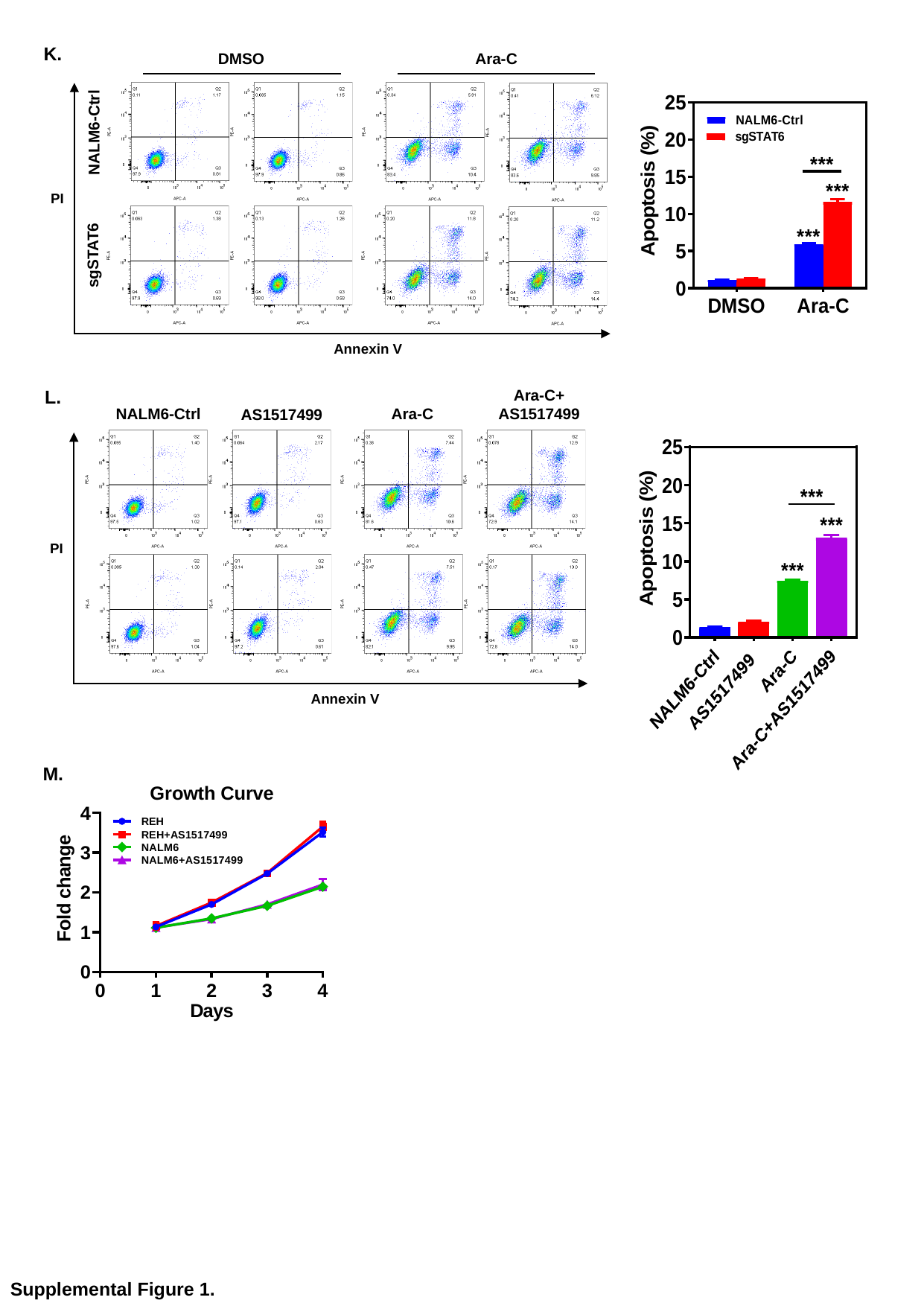

K.
Ara-C
DMSO
NALM6-Ctrl
PI
sgSTAT6
Annexin V
Ara-C+
AS1517499
Ara-C
NALM6-Ctrl
AS1517499
PI
Annexin V
L.
M.
Supplemental Figure 1.

## Slide 3
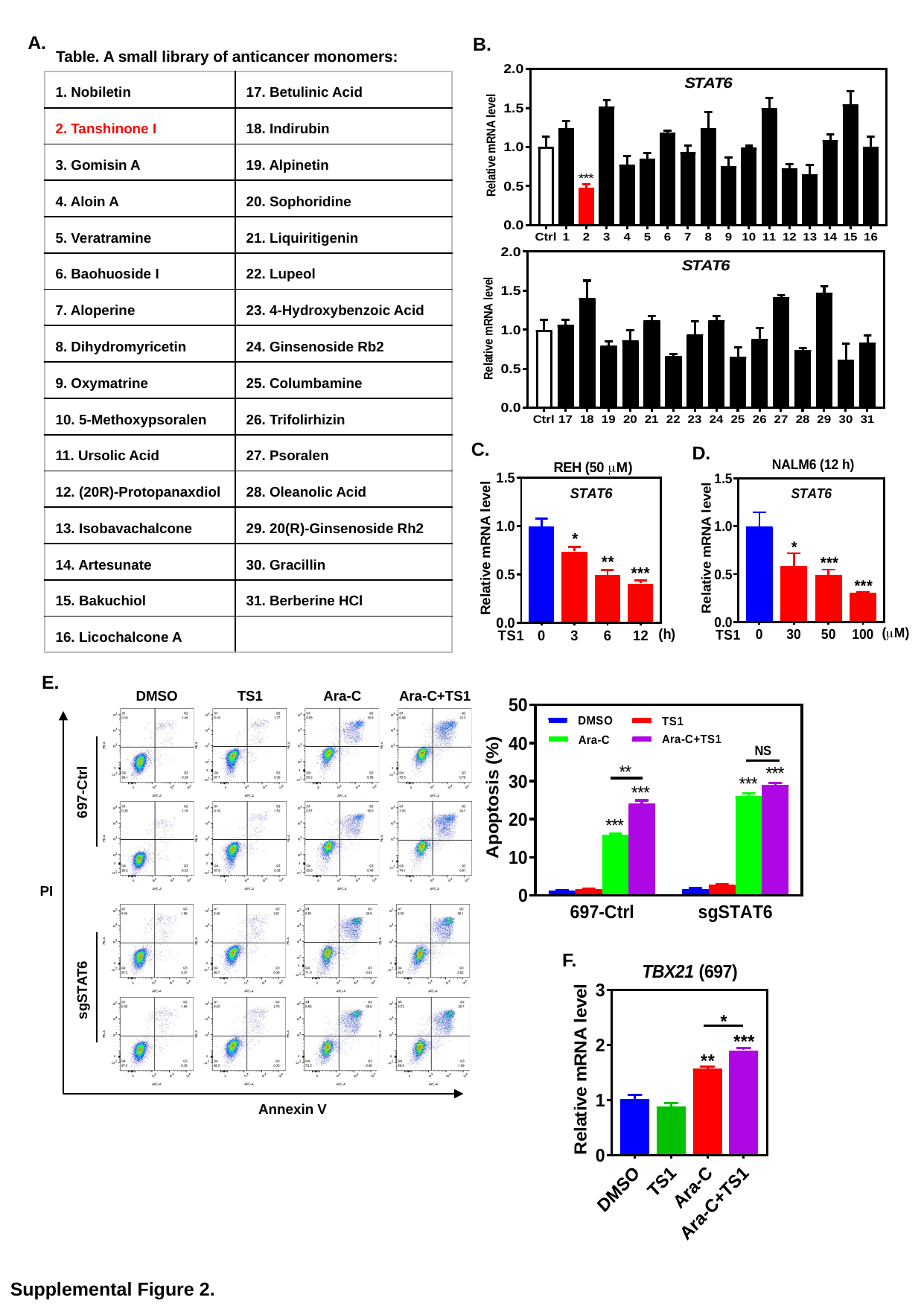

A.
B.
Table. A small library of anticancer monomers:
| 1. Nobiletin | 17. Betulinic Acid |
| --- | --- |
| 2. Tanshinone I | 18. Indirubin |
| 3. Gomisin A | 19. Alpinetin |
| 4. Aloin A | 20. Sophoridine |
| 5. Veratramine | 21. Liquiritigenin |
| 6. Baohuoside I | 22. Lupeol |
| 7. Aloperine | 23. 4-Hydroxybenzoic Acid |
| 8. Dihydromyricetin | 24. Ginsenoside Rb2 |
| 9. Oxymatrine | 25. Columbamine |
| 10. 5-Methoxypsoralen | 26. Trifolirhizin |
| 11. Ursolic Acid | 27. Psoralen |
| 12. (20R)-Protopanaxdiol | 28. Oleanolic Acid |
| 13. Isobavachalcone | 29. 20(R)-Ginsenoside Rh2 |
| 14. Artesunate | 30. Gracillin |
| 15. Bakuchiol | 31. Berberine HCl |
| 16. Licochalcone A | |
C.
D.
E.
DMSO
Ara-C
Ara-C+TS1
TS1
697-Ctrl
PI
sgSTAT6
Annexin V
F.
Supplemental Figure 2.

## Slide 4
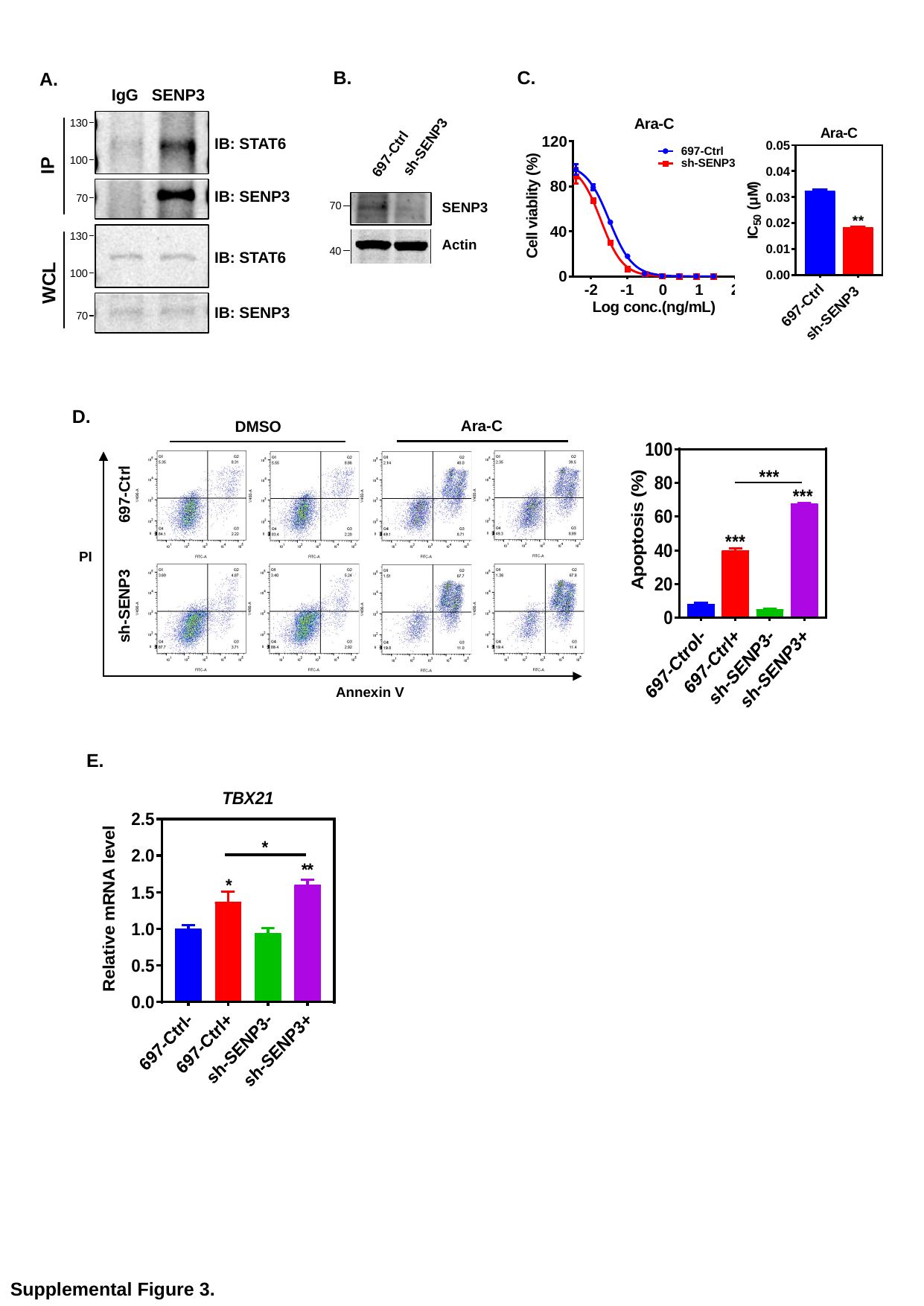

B.
C.
A.
IgG SENP3
130
IB: STAT6
100
IP
IB: SENP3
70
130
IB: STAT6
100
WCL
IB: SENP3
70
sh-SENP3
697-Ctrl
SENP3
70
Actin
40
D.
Ara-C
DMSO
697-Ctrl
PI
sh-SENP3
Annexin V
E.
Supplemental Figure 3.
