# Supplementary material for: Targeted deprivation of STAT6 sensitizes acute lymphoblastic leukemia cells to cytarabine in vivo and in vitro: clinical implications
Source: Cell Death Dis. 2025 Sep 2;16(1):669. doi: 10.1038/s41419-025-07981-7 (PMC12405434; doi:10.1038/s41419-025-07981-7)
Supplement: Supplementary file 2 — Supplementary Figure Legends [file 41419_2025_7981_MOESM2_ESM.docx]

**Supplemental Figure 1.**

(A) Gene expression of STAT2, STAT3, STAT4, STAT5a and STAT5b as determined by RNA-Seq analysis of 61 paired diagnosis-relapse specimens. Two sets of data are compared using two-tailed Student’s *t*-test, with *p*<0.05 as a statistically significant difference.

(B-F) Chemosensitivity of parental REH and STAT6-knockout REH cells to chemotherapeutic drugs commonly used in ALL treatment as determined by the cell viability assay.

(G) Endogenous STAT6 in NALM6 or 697 cells was knocked out using CRISPR-Cas9 technology.

(H) Chemosensitivity of parental NALM6 and STAT6-knockout NALM6 cells to Ara-C as determined by the cell viability assay.

(I) Chemosensitivity of parental 697 and STAT6-knockout 697 cells to Ara-C as determined by the cell viability assay.

(J) STAT6 knockout enhances Ara-C-induced apoptosis in REH cells. The DNA damage response and apoptosis biomarker were detected by WB analysis.

(K) The rate of apoptosis in parental NALM6 or STAT6-knockout NALM6 cells treated with Ara-C and analyzed by flow cytometry. The quantitative bar graph is shown on the right and data are depicted as mean ± SD, ****p*<0.001.

(L) Ara-C (20 ng/mL) induces apoptosis in NALM6 cells or NALM6 cells pretreated with AS1517499 (10 nM). The quantitative bar graph is shown on the right and data are presented as mean ± SD, each group is compared with the control using two-tailed Student’s *t*-test, ****p*<0.001.

(M) Growth curves of REH and NALM6 cells, as well as these cells pretreated with AS1517499.

**Supplemental Figure 2.**

(A) A small library of anticancer monomers.

(B) Effect of various TCM anticancer monomers (50 μM, 12 h) on the relative mRNA levels of *STAT6* in REH cells as determined by RT-qPCR. Data are presented as mean ± SD, two-tailed Student’s *t*-test, ****p*<0.001.

(C) Effect of TS1 (50 μM) on the relative mRNA levels of *STAT6* in REH cells at different time points. Each group is compared with the control using two-tailed Student’s *t*-test, **p*<0.05; ***p*<0.01; ****p*<0.001.

(D) Effect of increasing TS1 concentrations on the relative mRNA levels of *STAT6* in NALM6 cells treated for 12 h. Each group is compared with the control using two-tailed Student’s *t*-test, **p*<0.05; ****p*<0.001.

(E) The synergistic effect of TS1 on Ara-C-induced apoptosis in 697 cells is dependent on STAT6. The quantitative bar graph is shown on the right and data are presented as mean ± SD, two-tailed Student’s *t*-test, ****p*<0.001.

(F) Relative mRNA levels of *TBX21* in 697 cells and 697 cells pretreated with TS1 (10 mM, 48 h) under the treatment of Ara-C (20 ng/mL) as determined by RT-qPCR. Data are presented as mean ± SD, each group is compared with the control using two-tailed Student’s *t*-test, **p*<0.05; ***p*<0.01; ****p*<0.001.

**Supplemental Figure 3.**

(A) SENP3 binds to STAT6 in REH cells. Cell lysates were immunoprecipitated using an anti-SENP3 antibody followed by WB analysis.

(B) Endogenous SENP3 in 697 cells was knocked down using shRNA.

(C) SENP3 knockdown sensitizes 697 cells to Ara-C as determined by the cell viability assay. Data are presented as dose-response curve (left panel) and bar graph of IC_50_ values (right panel), two-tailed Student’s *t*-test, ***p*<0.01.

(D) SENP3 knockdown enhances Ara-C-induced apoptosis in 697 cells as determined by flow cytometry. Data are presented as mean ± SD and the quantitative bar graph is shown at right panel, two-tailed Student’s *t*-test, ****p*<0.001.

(E) Relative mRNA levels of *TBX21* in parental 697 and SENP3-knockdown 697 cells under Ara-C (20 ng/mL) treatment as determined by RT-qPCR. Data are presented as mean ± SD, two-tailed Student’s *t*-test, **p*<0.05; ***p*<0.01.
