# Supplementary figures and images for: Targeted deprivation of STAT6 sensitizes acute lymphoblastic leukemia cells to cytarabine in vivo and in vitro: clinical implications
Source: Cell Death Dis. 2025 Sep 2;16(1):669. doi: 10.1038/s41419-025-07981-7 (PMC12405434; doi:10.1038/s41419-025-07981-7)

Fig 1B.

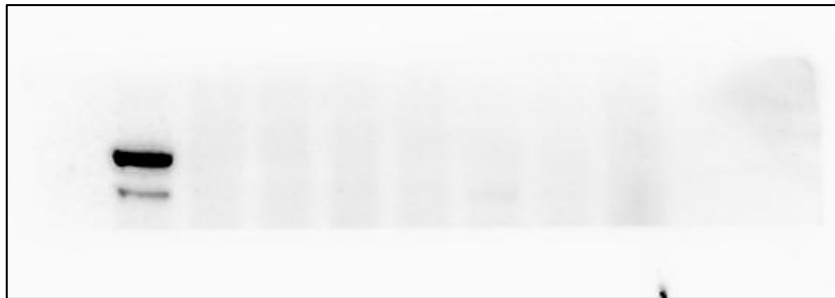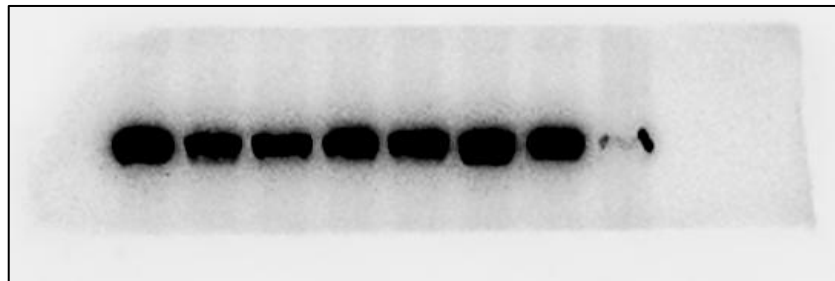

Fig 1G.

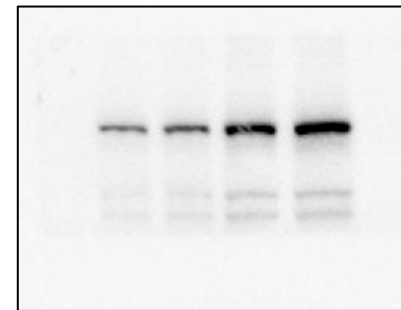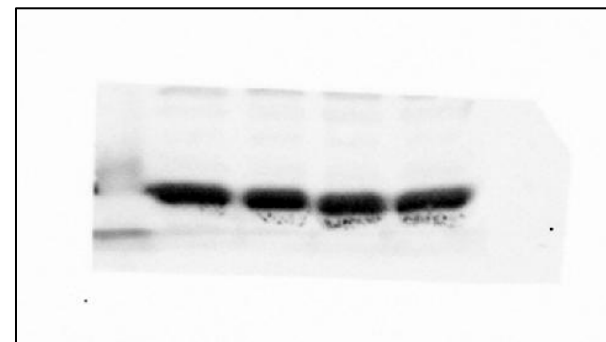

Fig 2C.

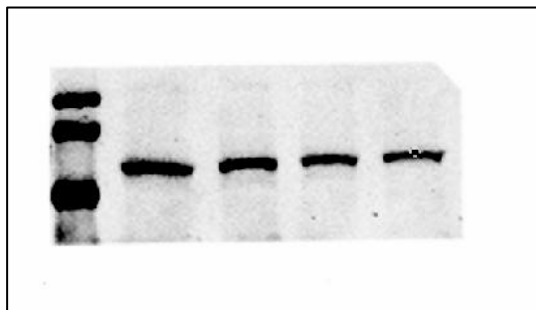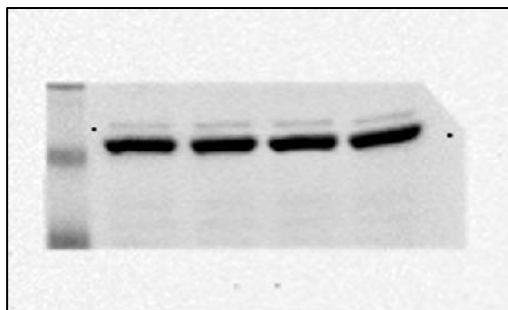

Fig 2E.

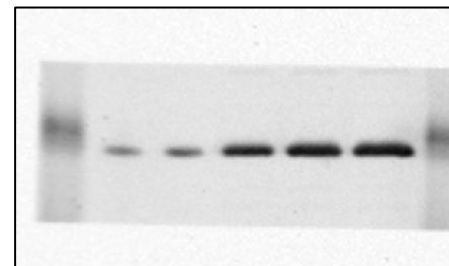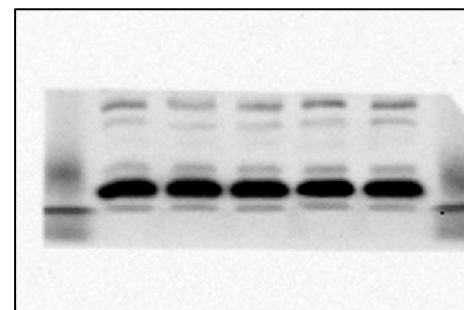

Fig 3A.

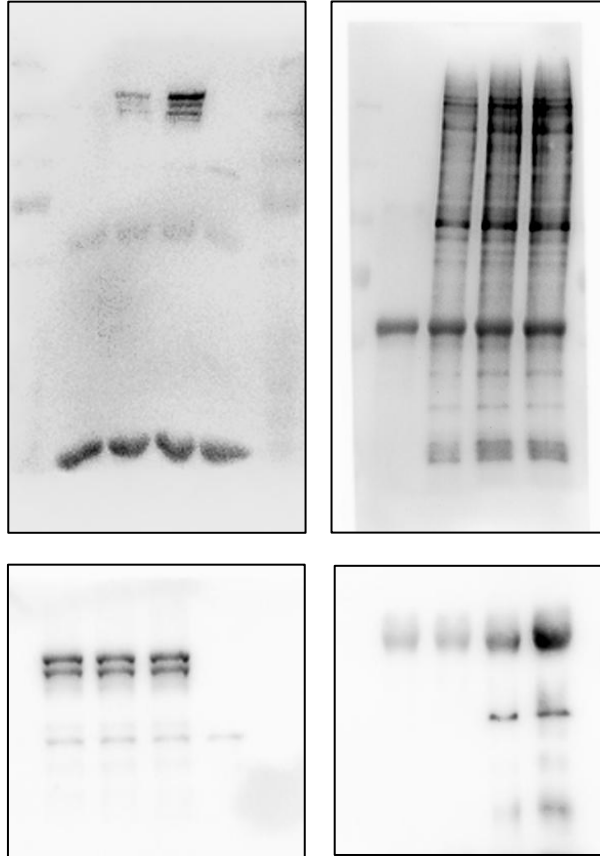

Fig 3B.

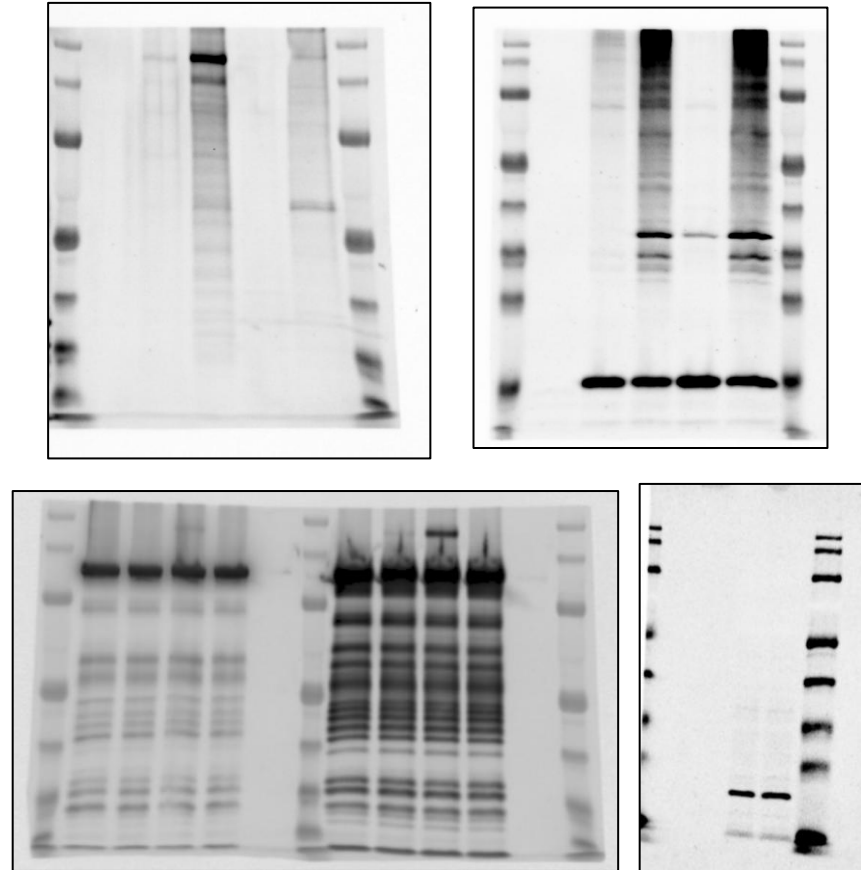

Fig 3C.

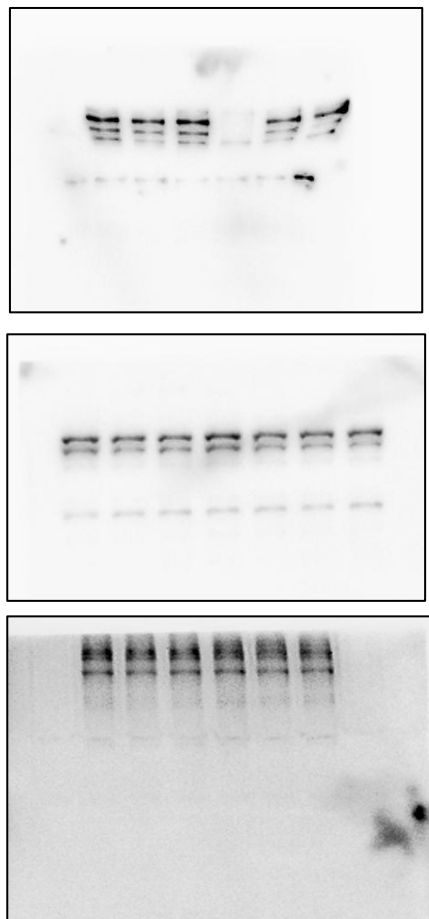

Fig 3D.

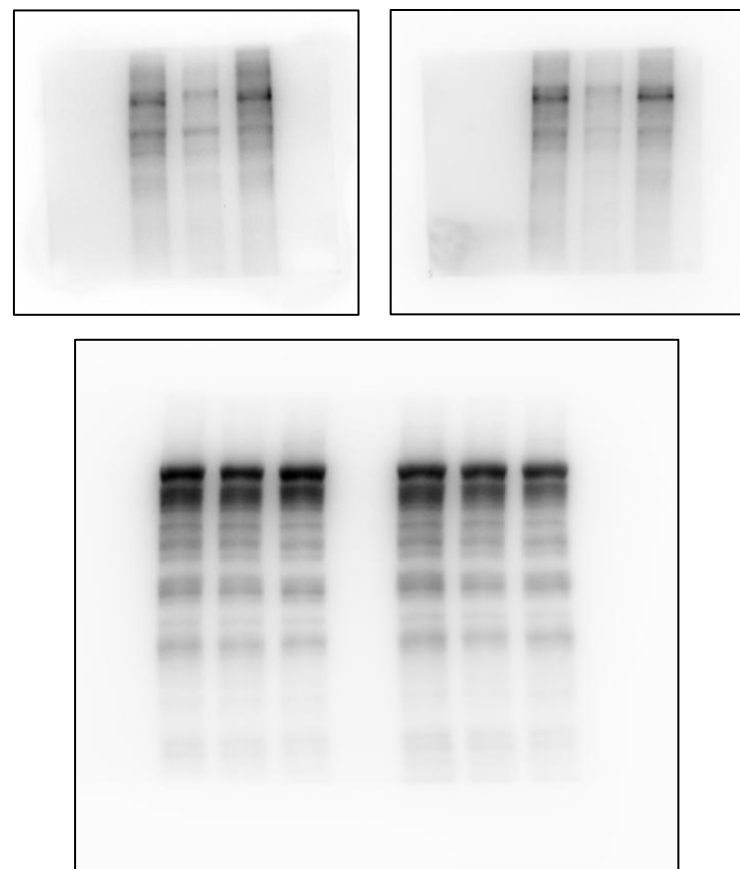

Fig 4A & 4B.

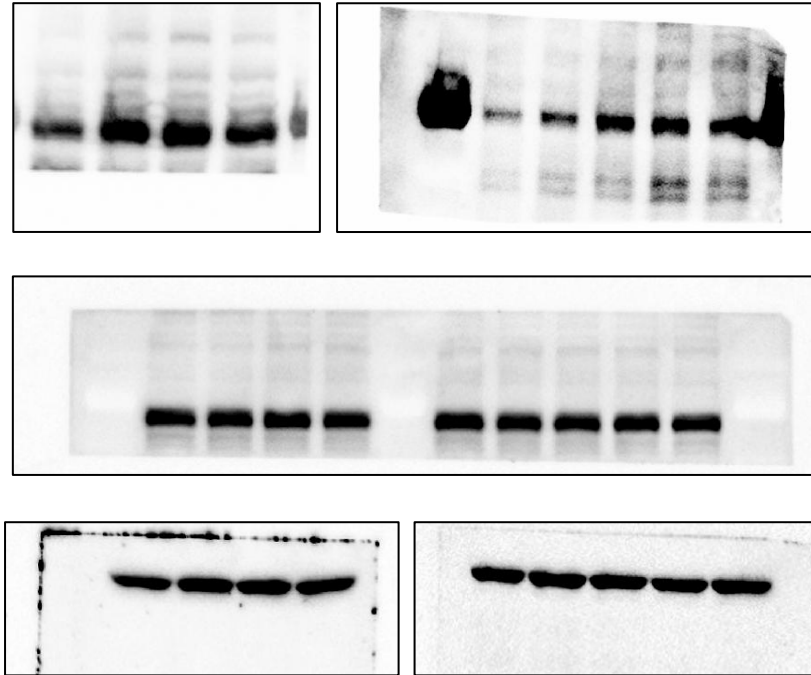

Fig 4C.

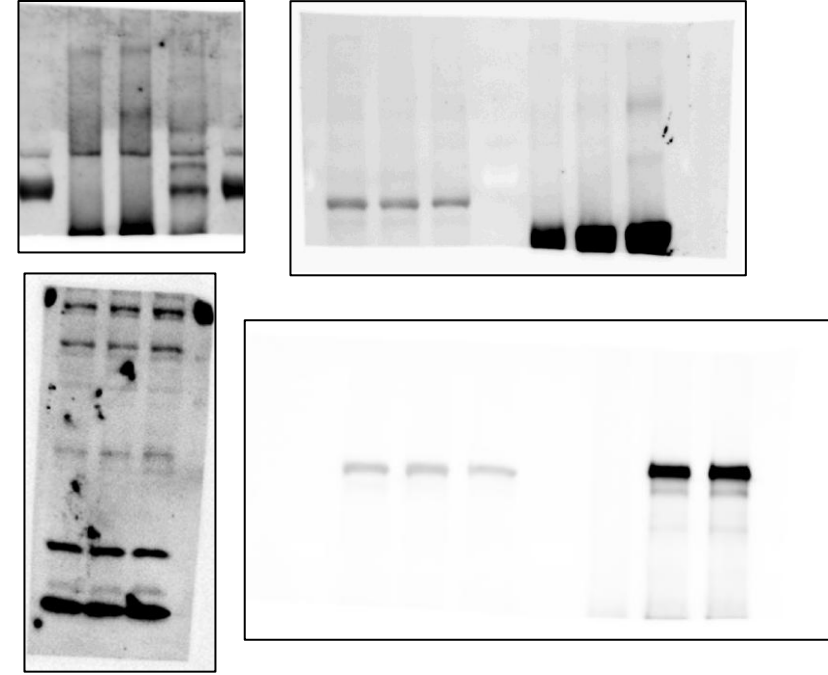

Fig 4D.

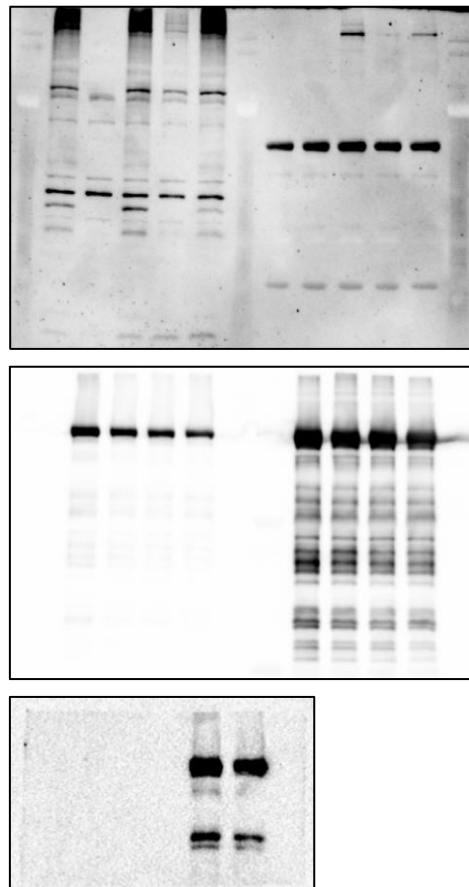

Fig 4E.

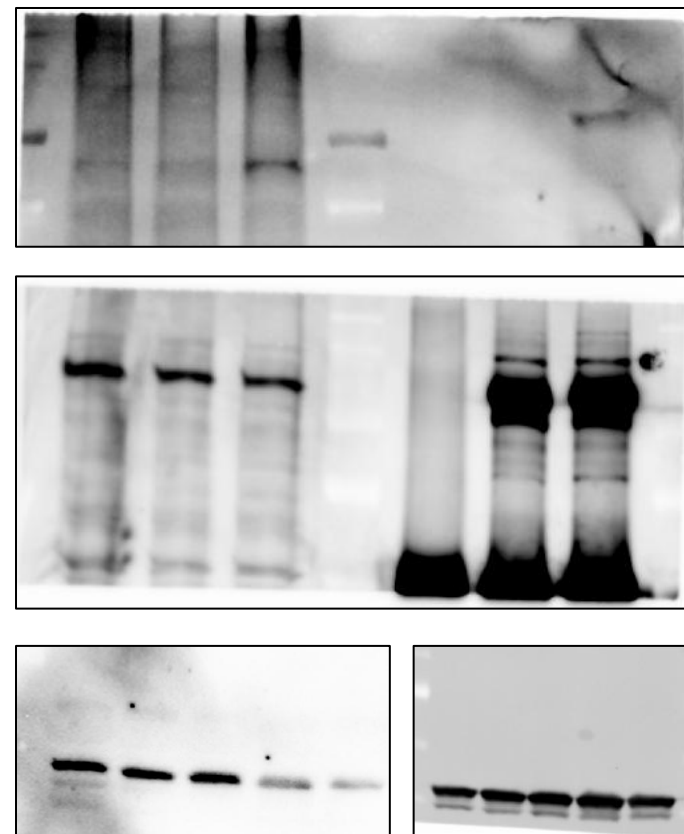

Fig S1H.

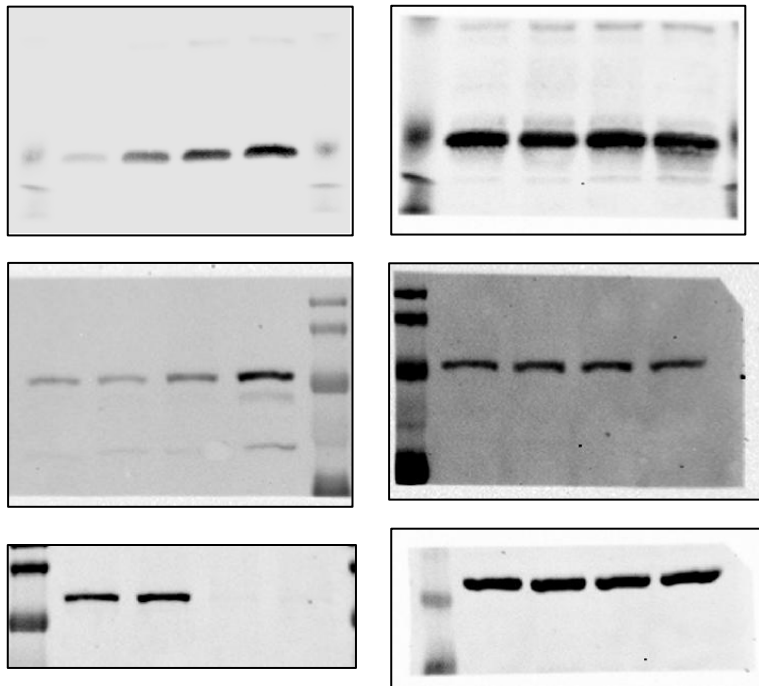

Fig S2E.

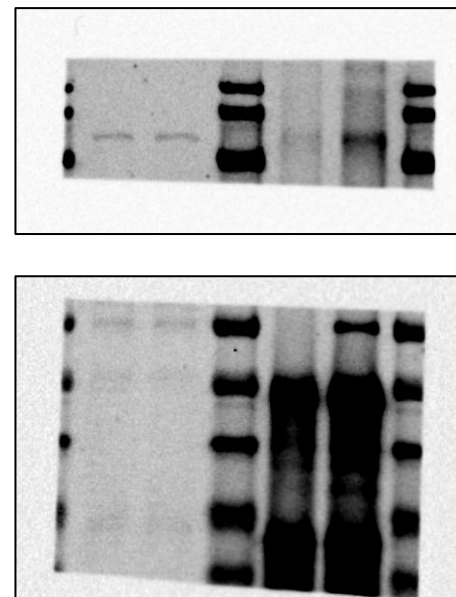

Supplement: Supplementary file 3 — Original Data [file 41419_2025_7981_MOESM3_ESM.pdf]
